# *SLC22A1* rs622342 Polymorphism Predicts Insulin Resistance Improvement in Patients with Type 2 Diabetes Mellitus Treated with Metformin: A Cross-Sectional Study

**DOI:** 10.1155/2020/2975898

**Published:** 2020-05-08

**Authors:** Kunrong Wu, Xiaoli Li, Yuedong Xu, Xiaoqian Zhang, Ziwan Guan, Shufang Zhang, Yan Li

**Affiliations:** ^1^Department of Clinical Pharmacy, The First Affiliated Hospital of Shandong First Medical University, Ji'nan 250014, China; ^2^School of Pharmaceutical Sciences, Shandong First Medical University, Tai'an 271000, China; ^3^Department of Endocrinology and Metabology, The First Affiliated Hospital of Shandong First Medical University, Ji'nan 250014, China

## Abstract

**Background:**

Metformin is the most widely used oral antidiabetic agent and can reduce insulin resistance (IR) effectively. Organic cation transporter 1 (encoded by *SLC22A1*) is responsible for the transport of metformin, and ataxia-telangiectasia-mutated (*ATM*) is a gene relating to the DNA repair and cell cycle control. The aim of this study was to evaluate if the genetic variants in *SLC22A1* rs622342 and *ATM* rs11212617 could be effective predictors of islet function improvement in patients with type 2 diabetes mellitus (T2DM) on metformin treatment.

**Methods:**

This cross-sectional study included 111 patients with T2DM treated with metformin. Genotyping was performed by the dideoxy chain-termination method. The homeostatic indexes of IR (HOMA-IR) and beta-cell function (HOMA-BCF) were determined according to the homeostasis model assessment.

**Results:**

Fasting plasma glucose (FPG) levels, HbA_1c_ levels, and HOMA-IR were significantly higher in patients with the rs622342 AA genotype than in those with C allele (*P* < 0.05). However, these significant differences were not observed between rs11212617 genotype groups. Further data analysis revealed that the association between the rs622342 polymorphism and HOMA-IR was gender related, and so was rs11212617 polymorphism and HOMA-BCF. HOMA-IR was significantly higher in males with rs622342 AA genotype than in those with C allele (*P*=0.021), and HOMA-BCF value was significantly higher in females carrying rs11212617 CC genotype than in those with A allele (*P*=0.038). The common logarithm (Lg10) of HOMA-BCF was positively correlated with the reciprocal of HbA_1c_ (*r* = 0.629, *P* < 0.001) and negatively associated with Lg10 FPG (*r* = −0.708, *P* < 0.001).

**Conclusions:**

The variant of rs622342 could be a predictor of insulin sensitivity in patients with T2DM treated with metformin. The association between the rs622342 polymorphism and HOMA-IR and the association between the rs11212617 polymorphism and HOMA-BCF were both gender related.

## 1. Introduction

The decline of islet function is one of the most important determinants in the occurrence and development of type 2 diabetes mellitus (T2DM). Therefore, to assess the factors influencing islet function is of great significance for improving the prognosis of patients with T2DM. The evaluation of islet function includes insulin sensitivity and *β*-cell secretion. Insulin resistance (IR), which reflects insulin sensitivity, can be described as a decreased response of muscle and adipose tissues to the effects of insulin [1]. IR is associated with an increasing risk of developing T2DM and cardiovascular disease [2]. The gold standard for IR and *β*-cell secretion evaluation is the hyperinsulin-euglycemic clamp test [3]. However, this method is too complex to be widely carried out in clinical practice. HOMA-IR and HOMA-BCF are parameters of the homeostasis model, which are determined by fasting blood glucose and fasting insulin levels, and are more suitable for clinical evaluation of insulin sensitivity and *β*-cell function [4].

Metformin is a widely used insulin sensitizer and a first-line treatment for T2DM. It is associated with enhanced insulin sensitivity, improved IR, and reduced risk of cardiovascular complications in patients with diabetes [5–7]. Metformin is a synthetic biguanide which belongs to a hydrophilic base, existing in cationic form at physiological pH and with a minimal passive diffusion of the membrane owning to the polar guanidine fraction [8]. Organic cation transporters (OCTs) are one of the important transporter families of metformin [9]. OCT1 is a member of the OCT family and encoded by *SLC22A1* gene located at chromosome 6q25.3. It is primarily expressed in the apical membrane of kidney cells and basolateral membrane of hepatocytes and intestinal cells and is responsible for the renal transport, hepatic uptake, and intestinal absorption of metformin [9–12]. Ataxia-telangiectasia-mutated (*ATM*) gene belongs to the phosphatidylinositol 3 kinase-related kinase family and locates at the long arm of chromosome 11q22-23. A genome-wide association study (GWAS) suggested that the top single nucleotide polymorphism rs11212617 in *ATM* is associated with metformin response [13]. Nevertheless, the Diabetes Prevention Program (DPP) study did not support the association of the C allele at rs11212617 with improved metformin action on glycemic control [14].

Previous studies have reported the association between variations in *SLC22A1* rs622342 or *ATM* rs11212617 and the glycemic response to metformin [13, 15, 16], although consistent results have not been obtained yet [13–19]. However, little is known about the role of the variants at the two loci in the IR or *β*-cell function in patients with T2DM on metformin treatment. Therefore, we designed this cross-sectional study mainly to evaluate whether the genetic variants in rs622342 and rs11212617 were associated with the *β*-cell function (HOMA-BCF) and IR (HOMA-IR) and explore the predictive role of rs622342 and rs11212617 polymorphisms in the individual difference of islet functions in patients with T2DM treated with metformin.

## 2. Materials and Methods

### 2.1. Study Subjects and Data Collection

We designed a cross-sectional study by reviewing the electronic medical database of patients. The sample size was calculated using PASS 11.0 software. In this study, we enrolled 111 patients with T2DM totally (World Health Organization 1999 criteria). All participants were genetically unrelated Chinese and recruited from the Endocrinology Department, Shandong Provincial Qianfoshan Hospital from April 2018 to May 2019. Subjects were receiving metformin at least 6 months, which could be identified by searching the medical record database for each patient enrolled. The dose range of metformin was 1000 mg to 2500 mg per day (there is no statistical difference of metformin daily dose (mg/day) among males and females). None of the subjects were receiving insulin and insulin secretagogues therapy. Patients with hyperthyroidism, hypothyroidism, serious renal failure, chronic hepatic diseases, malignant diseases (such as cancer), autoimmune diseases, pregnancy, or lactation were excluded. The study was approved by the local Medical Ethics Committee and adhered to the principles of the Helsinki Declaration of 1975. Informed consent of the study was obtained from all subjects upon recruitment.

We collected demographic data and laboratory results including triglyceride (TG), serum creatinine (Scr), total cholesterol (TC), fasting plasma glucose (FPG), alanine aminotransferase (ALT), aspartate aminotransferase (AST), low-density lipoprotein cholesterol (LDL-C), high-density lipoprotein cholesterol (HDL-C), C-peptide, HbA_1c_ levels, and insulin concentrations from the clinical sources. We used HOMA-IR to assess insulin sensitivity and HOMA-BCF to assess *β*-cell function. HOMA-IR and HOMA-BCF were calculated according to the homeostasis model of assessment as the following equations [20, 21]:

(1) $$\begin{array}{c} \text{HOMA}-\text{IR}=\frac{\text{fasting glucose}\left(\text{mmol/L}\right)\times \text{fasting insulin}\left(\mu \text{U/mL}\right)}{22\text{.}5}\text{,}\\ \begin{array}{c} \text{HOMA}-\text{BCF}=\frac{20\times \text{fasting insulin}\left(\mu \text{U/mL}\right)}{\text{fasting glucose}\left(\text{mmol/L}\right)\text{ –}3\text{.}5} \end{array}\text{.} \end{array}$$

### 2.2. Genotyping

A 2-ml whole fresh blood sample was collected from each patient in an EDTA tube for genotyping. DNA was extracted from peripheral blood samples using the Tiangen Blood DNA Kit (Tiangen Inc., Beijing, China). *SLC22A1* rs622342 and *ATM* rs11212617 variants were analyzed by the dideoxy chain-termination method. The amplification primers used for polymerase chain reaction (PCR) were as follows: F 5′-CACCACATGAGTTAACAGCAGATT-3′ and R 5′-GCTCAAGCAAGCCTCCTACC-3′ for *SLC22A1* rs622342; F 5′-CTCAATTAAAACCAGAGAAGGCAG-3′ and R 5′-AATTTTTTGCGTGGAGTCAGAGTC-3′ for *ATM* rs11212617. A 25 *μ*l PCR mixture reaction system included 1 *μ*l upstream primer (3.2 pmol/*μ*L), 1 *μ*l downstream primer (3.2 pmol/*μ*L), 0.25 *μ*l 2 × Taq DNA polymerase, 0.5 *μ*l dNTP mixture, 1 *μ*l DNA, and ddH_2_O 21.25 *μ*l. PCR reaction conditions included initial predenaturation at 95°C for 5 min, followed by 40 cycles of denaturation for 30 s at 95°C, annealing for 30 s at 58°C, extension for 1 min at 72°C, with a final extension at 72°C for 5 min. The PCR product was electrophoresed by an ABI3730XL (Applied Biosystems, Inc., Carlsbad, California, America) sequencer after purified.

### 2.3. Statistical Analyses

All data were analyzed by using SPSS (version 22.0, SPSS Inc., Chicago, IL, USA) software. Parameter variables were presented as mean ± standard deviation, and categorical variables were expressed as numbers (percentage). The Kolmogorov–Smirnov test was used to verify the normal distribution of continuous variables. Comparisons difference between groups by the ANOVA or *t*-test for continuous variables and the chi-square test for discrete variables. Furthermore, a linear regression model was used to assess the effects of genetic polymorphisms on HOMA-IR and HOMA-BCF, and the data were adjusted for potential confounders. The association between study variables was evaluated by Pearson correlation coefficient. Results were considered statistically significant for two-tailed *P* value less than 0.05. The chi-square test was used to validate the Hardy–Weinberg equilibrium. Value of *P* > 0.05 indicated that the genotype frequencies of the samples were consistent with Hardy–Weinberg equilibrium.

## 3. Results

### 3.1. Clinical Parameters of the Subjects

Although 111 patients were enrolled totally in this study, a total of 101 test results for *SLC22A1* rs622342 and 104 for *ATM* rs11212617 were obtained due to undetectable blood samples. The distribution of *SLC22A1* rs622342 (*P*=0.767) and *ATM* rs11212617 (*P*=0.587) genotypes and alleles followed the Hardy–Weinberg equilibrium in this study subjects. As shown in Table 1, the groups did not differ statistically in age, SBP, DBP, ALT, AST, TC, HDL-C, LDL-C, gender, duration, weight, BMI, insulin, and C-peptide, while the values of TG, Scr, FPG, and HbA_1c_, were significantly higher in patients carrying the rs622342 AA genotype than in those with the AC/CC genotype (*P* < 0.05). HOMA-IR, calculated from fasting blood glucose and fasting insulin, showed significant differences between rs622342 AA and C allele carriers (*P*=0.004). After adjustment for gender, duration, BMI, FPG, HbA_1c_, and insulin, the difference in HOMA-IR between the two groups remained significant (*P*^*adj*^=0.025). In terms of *β*-cell function, patients with the AA genotype had lower HOMA-BCF than those with the AC/CC genotype; nonetheless, the differences were not statistically significant (*P*^*adj*^=0.312).

There were no significant differences between *ATM* rs11212617 CC and AA/AC genotypes with respect to age, SBP, DBP, ALT, AST, TG, TC, HDL-C, LDL-C, Scr, duration, weight, BMI, FPG, HbA_1c_, insulin, or C-peptide. Interestingly, we found the frequency of AC/AA genotypes was higher in males than in females (*P*=0.020). HOMA-IR and HOMA-BCF values were higher in patients with the CC than those with the AA/AC. However, these differences did not reach statistical significance after adjustment for the covariates (Table 2).

### 3.2. HOMA-BCF and HOMA-IR among the Genotypes of SLC22A1 rs622342 and ATM rs11212617 according to Gender, BMI, and HbA_1c_

As shown in Table 3, the association between the rs622342 polymorphism and HOMA-IR was gender-related, and so was the association between the rs11212617 polymorphism and HOMA-BCF. HOMA-IR was significantly higher in males with rs622342 AA than in those with the AC/CC genotype (*P* = 0.021), while we did not find significant difference among females (*P* = 0.118). HOMA-BCF was significantly higher in females with rs11212617 CC than in those with the AA/AC genotype (*P* = 0.038). Such significant difference was not observed in males. Among subjects with BMI < 25, there was extremely significant difference between the HOMA-IR in patients carrying the rs622342 AA genotype and in those with the AC/CC genotype (*P* = 0.002). Among subjects with HbA_1c_ ≥ 8%, HOMA-IR in patients carrying the rs622342 AA genotype was much higher than in those with the AC/CC genotype (*P* = 0.010). Nevertheless, there was no significant difference in HOMA-BCF between the genotypes of rs11212617 according to BMI and HbA_1c._ Moreover, no significant associations were found between HOMA-BCF and rs622342 variants according to gender, BMI, and HbA_1c_, and the associations between HOMA-IR and rs11212617 variants were also not significant according to gender, BMI, and HbA_1c_.

### 3.3. Correlation between HOMA-BCF, Fasting Glucose, and HbA_1c_

Linear regression analysis found that the common logarithm (Lg) of HOMA-BCF was positively correlated with the reciprocal of HbA_1c_ (*r* = 0.629, *P* < 0.001) and negatively associated with Lg FPG (*r* = −0.708, *P* < 0.001).

## 4. Discussion

The current study found that the gene mutation in *SLC22A1* rs622342 was associated with lower risk of IR in patients with T2DM on metformin treatment. Patients with the rs622342C allele (minor allele) had significantly lower HOMA-IR value than those with the common genotype (AA). However, this significant difference was not observed in *ATM* rs11212617 polymorphisms. Furthermore, we found no association between HOMA-BCF and rs622342 or rs11212617 polymorphisms in present study subjects. These results may imply that the C allele of *SLC22A1* rs622342 was associated with a lower risk of IR in patients with type 2 diabetes on metformin treatment. Our findings are partially consistent with those obtained by Berstein et al. [17], which indicated that patients carrying the rs622342 CC genotype had significantly lower HOMA-IR than those carrying the AC/AA genotype, whereas there was no significant difference in HOMA-IR between different genotypes of rs11212617. To the best of our knowledge, this is the first study to evaluate this association in patients with T2DM treated with metformin in Chinese population. There have been very few experiments to report such findings till now. One study conducted by Berstein et al. [17] indicated that HOMA-IR was significantly higher in patients with the OCT1–R61C CC genotype than in those with the CT/TT genotype. Another study [4] showed that patients with the *OCT2*–T201M CT/TT genotype had significantly higher HOMA-IR than those with the CC genotype.

In the present study, patients with the rs622342C allele were associated with better control of FPG and HbA_1c_. This may be due to the different affects of the rs622342 variant on metformin glycemic response. However, we did not find any association between the rs11212617 variant and the levels of HbA_1c_ and FPG. It is possible that there was no difference in the effect of rs11212617 mutation on metformin glycemic response. Previous studies had confirmed that the genetic variation of rs622342 and rs11212617 was associated with metformin glycemic response. Becker et al. [16] reported that rs622342 genetic variation was associated with the glucose-lowering effect of metformin in patients with T2DM, and for each minor C allele, the decrease in HbA_1c_ levels was 0.28% less. Moreover, A GWAS study established in UK population suggested that the top SNP rs11212617C allele in *ATM* is associated with greater metformin response [13]. Conversely, the DPP study reported that the rs11212617C allele did not associate with metformin glycemic response [14]. Furthermore, another study conducted by Berstein et al. [17] showed that both *OCT1* rs622342 and *ATM* rs11212617 polymorphisms were not associated with HbA1c levels in patients treated with metformin. These inconsistent results may be due to several reasons. First, the clinical response to metformin was related to gender and BMI. Second, metformin may be more effective in patients with a higher HbA_1c_ levels at baseline, and therefore the curative effects of genotype on response of metformin may be easier to detect in the disease setting. Third, geographic and ethnic factors may emphasize this phenomenon.

In this study, further analysis showed that the association between the rs622342 polymorphism and HOMA-IR and the association between the rs11212617 polymorphism and HOMA-BCF were both gender related. HOMA-IR was significantly higher in males with the rs622342 AA genotype than in those with the C allele, while in females the difference did not reach significance. Moreover, HOMA-BCF was significantly higher in females carrying the rs11212617 CC genotype than in those carrying the AA/AC genotype. Such significant difference was not observed in males. These findings are partially according with the previous study, which showed that the association between the *OCT2*-T201M variant and HOMA-IR and HOMA-BCF was associated with males [4].

The common logarithm (Lg) of HOMA-BCF was positively related with the reciprocal of HbA_1c_ and negatively correlated with Lg FPG in this study. These findings are consistent with those obtained by previous studies [4, 22], which showed that HOMA-BCF was inversely associated with fasting glucose and HbA_1c_. These results indicated that when BCF increases, FPG and HbA1c concentrations decrease. However, HOMA-BCF in patients carrying at least one C allele (*SLC22A1* rs622342) was higher than in those carrying the AA genotype. Conversely, insulin and C-peptide levels were higher in *SLC22A1* rs622342 AA genotype carriers than in the C allele carriers. This means that the compensatory process for insulin secretion in response to elevated FPG levels was not sufficient in *SLC22A1* rs622342 AA genotype carriers.

This is a cross-sectional study to evaluate the association between HOMA-IR and HOMA-BCF and genetic variants in rs622342 and rs11212617 in patients with T2DM on metformin treatment. Taken our results together, metformin therapy may be more effective in patients carrying the rs622342C allele in this study subjects. Furthermore, the association between the rs622342 polymorphism and IR varies among genders, and so was the rs11212617 polymorphism and BCF. This suggested that the effect of genetic polymorphisms on the efficacy of metformin needs to consider gender factors. Our findings may be valuable for the clinical individualized treatment of metformin in patients with T2DM in Chinese. However, due to the difference of the effect of genetic polymorphisms on metformin efficacy in diabetic patients, clinical studies that including larger sample size are still required to further validate these results.

The main limitation of the present study is the relative small sample size. We used the minimum sample size estimated by PASS 11.0 software, but more reliable results could be obtained if we expanded the sample size. Another limitation is the lack of information on compliance/adherence of the patients and lifestyle information, which could affect the glycemic control and response to hypoglycemic agents.

## 5. Conclusion

*SLC22A1* rs622342C allele associated with lower IR index in patients with T2DM treated with metformin, but not associated with BCF, and could be predicting IR improvement in patients with T2DM treated with metformin. The association between rs622342 polymorphism and HOMA-IR and the association between rs11212617 polymorphism and HOMA-BCF were both gender related. Studies that including larger sample size are still required to further evaluation on the role of *SLC22A1* rs622342 variants on IR improvement in patients with T2DM on metformin treatment.

## Figures and Tables

**Table 1:** Clinical characteristics of the subjects according to the genotypes of *SLC22A1* rs622342.

Parameter	*SLC22A1* rs622342			
	AA (*n* = 60)	AC + CC (*n* = 41)	*P* value^*∗*^	*P* ^*adj*^ value^*∗*^
Age	58.32 ± 10.89	57.07 ± 9.32	0.556	—
Systolic blood pressure (mmHg)	129.38 ± 12.61	128.98 ± 11.45	0.870	—
Diastolic blood pressure (mmHg)	77.72 ± 8.55	78.34 ± 9.09	0.747	—
Alanine aminotransferase (U/L)	24.19 ± 18.78	20.21 ± 9.93	0.597	—
Aspartate aminotransferase (U/L)	21.89 ± 14.82	17.72 ± 4.66	0.860	—
Triglyceride (mmol/L)	1.76 ± 1.02	1.48 ± 0.92	0.040	—
Total cholesterol (mmol/L)	4.55 ± 1.03	4.34 ± 1.06	0.335	—
HDL-C (mmol/L)	1.20 ± 0.33	1.26 ± 0.36	0.202	—
LDL-C (mmol/L)	2.60 ± 0.71	2.38 ± 0.83	0.172	—
Serum creatinine (mg/dL)	61.27 ± 14.39	67.20 ± 13.33	0.028	—
Male/female	32/28 (53.3/46.7)	29/12 (70.7/29.3)	0.079^$^	—
Duration (years)	7.79 ± 4.24	9.81 ± 6.05	0.158	—
Weight (kg)	72.60 ± 11.42	73.50 ± 12.18	0.557	—
BMI (kg/m^2)^	25.84 ± 3.05	25.87 ± 3.33	0.960	—
Fasting plasma glucose (mmol/L)	8.84 ± 2.88	7.59 ± 1.97	0.014	—
HbA_1c_ (%) (mmol/mol)	8.63 ± 1.98	7.83 ± 1.41	0.046	—
Insulin (*μ*U/mL)	9.34 ± 5.80	7.44 ± 4.73	0.067	—
C-peptide (ng/mL)	2.42 ± 1.01	2.07 ± 0.93	0.066	—
HOMA-IR	3.54 ± 2.27	2.38 ± 1.44	0.004	0.025
HOMA-BCF	48.54 ± 46.54	51.52 ± 51.67	0.936	0.312

HOMA-IR : *R* squared = 0.874 (adjusted *R* squared = 0.865)				
HOMA-BCF : *R* squared = 0.882 (adjusted *R* squared = 0.873)				

Data are presented as mean ± standard deviation, or *n* (%). ^*∗*^*P* value with ANOVA. ^$^*P* value with the chi-square test. ^adj^*P* value after adjustment for gender, duration, BMI, and levels of fasting plasma glucose, HbA_1c_, and insulin. LDL-C, low-density lipoprotein cholesterol; HDL-C, high-density lipoprotein cholesterol; HOMA-IR, homeostasis model assessment-insulin resistance; HOMA-BCF, homeostasis model assessment-beta-cell function.

**Table 2:** Clinical characteristics of the subjects according to the genotypes of *ATM* rs11212617.

Parameter	*ATM* rs11212617			
	AC + AA (*n* = 55)	CC (*n* = 49)	*P* value^*∗*^	*P* ^adj^ value^*∗*^
Age	56.42 ± 10.40	57.92 ± 9.73	0.455	—
Systolic blood pressure (mmHg)	129.33 ± 12.63	129.29 ± 11.73	0.978	—
Diastolic blood pressure (mmHg)	78.69 ± 8.49	77.73 ± 9.96	0.584	—
Alanine aminotransferase (U/L)	23.32 ± 15.38	21.16 ± 12.64	0.521	—
Aspartate aminotransferase (U/L)	18.99 ± 9.31	20.12 ± 9.47	0.504	—
Triglyceride (mmol/L)	1.83 ± 1.06	1.51 ± 0.76	0.080	—
Total cholesterol (mmol/L)	4.64 ± 1.09	4.34 ± 0.99	0.155	—
HDL-C (mmol/L)	1.22 ± 0.35	1.23 ± 0.33	0.665	—
LDL-C (mmol/L)	2.64 ± 0.74	2.44 ± 0.78	0.189	—
Serum creatinine (mg/dL)	63.38 ± 14.49	64.10 ± 14.29	0.771	—
Male/female	35/14 (71.4/28.6)	27/28 (49.1/50.9)	0.020^$^	—
Duration (years)	8.24 ± 5.21	8.62 ± 5.38	0.707	—
Weight (kg)	72.85 ± 12.32	73.80 ± 11.11	0.685	—
BMI (kg/m^2)^	25.84 ± 2.97	25.81 ± 3.12	0.961	—
Fasting plasma glucose (mmol/L)	8.35 ± 2.30	8.43 ± 3.09	0.924	—
HbA_1c_ (%) (mmol/mol)	8.40 ± 1.81	8.24 ± 1.89	0.496	—
Insulin (*μ*U/mL)	7.65 ± 3.94	9.77 ± 6.40	0.181	—
C-peptide (ng/mL)	2.10 ± 0.81	2.47 ± 1.08	0.117	—
HOMA-IR	2.76 ± 1.50	3.71 ± 3.34	0.215	0.244
HOMA-BCF	44.85 ± 45.79	54.05 ± 46.51	0.290	0.691

HOMA-IR : *R* squared = 0.874 (adjusted *R* squared = 0.865)				
HOMA-BCF : *R* squared = 0.879 (adjusted *R* squared = 0.870)				

Data are presented as mean ± standard deviation, or *n* (%). ^*∗*^*P* value with ANOVA. ^$^*P* value with the chi-square test. ^adj^*P* value after adjustment for gender, duration, BMI, and levels of fasting plasma glucose, HbA_1c_, and insulin. *ATM*, ataxia-telangiectasia-mutated; LDL-C, low-density lipoprotein cholesterol; HDL-C, high-density lipoprotein cholesterol; HOMA-IR, homeostasis model assessment-insulin resistance; HOMA-BCF, homeostasis model assessment-beta-cell function.

**Table 3:** The distributions of HOMA-IR and HOMA-BCF among the genotypes of *SLC22A1* rs622342 and *ATM* rs11212617 according to gender, BMI, and HbA_1c_.

	*SLC22A1* rs622342	HOMA-IR	HOMA-BCF	*ATM* rs11212617	HOMA-IR	HOMA-BCF
Male	AA (*n* = 32)	3.34 ± 2.21	41.61 ± 30.43	AA + AC (*n* = 35)	2.78 ± 1.57	50.88 ± 52.88
	AC + CC (*n* = 29)	2.36 ± 1.44	46.91 ± 51.51	CC (*n* = 27)	3.21 ± 2.41	44.34 ± 32.58
	*P* value	0.021	0.906	*P* value	0.598	0.718

Female	AA (*n* = 28)	3.77 ± 2.32	56.45 ± 58.86	AA + AC (*n* = 20)	2.72 ± 1.38	34.29 ± 26.41
	AC + CC (*n* = 12)	2.42 ± 1.42	62.66 ± 50.35	CC (*n* = 22)	4.33 ± 4.14	65.97 ± 57.08
	*P* value	0.118	0.586	*P* value	0.254	0.038

BMI ≥ 25	AA (*n* = 35)	4.20 ± 2.48	54.31 ± 49.20	AA + AC (*n* = 35)	3.17 ± 1.51	48.23 ± 48.97
	AC + CC (*n* = 28)	2.92 ± 1.42	62.73 ± 57.66	CC (*n* = 30)	4.32 ± 3.89	56.19 ± 46.42
	*P* value	0.025	0.885	*P* value	0.405	0.256

BMI < 25	AA (*n* = 25)	2.60 ± 1.50	40.46 ± 41.18	AA + AC (*n* = 20)	2.03 ± 1.16	38.92 ± 38.92
	AC + CC (*n* = 13)	1.21 ± 0.38	27.43 ± 20.15	CC (*n* = 19)	2.75 ± 1.86	50.68 ± 46.46
	*P* value	0.002	0.609	*P* value	0.262	0.660

HbA_1c_ ≥ 8%	AA (*n* = 33)	3.38 ± 2.24	32.43 ± 38.45	AA + AC (*n* = 32)	2.60 ± 1.70	22.84 ± 15.15
	AC + CC (*n* = 16)	1.89 ± 1.33	23.81 ± 19.65	CC (*n* = 21)	3.78 ± 4.29	29.94 ± 22.39
	*P* value	0.010	0.506	*P* value	0.432	0.604

HbA_1c_ < 8%	AA (*n* = 27)	3.73 ± 2.30	68.22 ± 47.97	AA + AC (*n* = 23)	2.98 ± 1.13	75.46 ± 55.53
	AC + CC (*n* = 25)	2.69 ± 1.42	69.26 ± 57.67	CC (*n* = 28)	3.67 ± 2.40	72.13 ± 51.45
	*P* value	0.048	0.541	*P* value	0.568	0.649

Data are given as mean ± standard deviation and are tested by Student's *t*-test. HOMA-IR, homeostasis model assessment-insulin resistance; HOMA-BCF, homeostasis model assessment-beta-cell function; *ATM*, ataxia-telangiectasia-mutated; BMI, body mass index.

## Data Availability

The clinical data used to support the findings of this study are available from the corresponding author upon request.
